# Supplementary material for: Longitudinal multi-omics analysis of host microbiome architecture and immune responses during short-term spaceflight
Source: Nat Microbiol. 2024 Jun 11;9(7):1661–75. doi: 10.1038/s41564-024-01635-8 (PMC11222149; doi:10.1038/s41564-024-01635-8)
Supplement: Supplementary file 1 — Supplementary Figs. 1–7. [file 41564_2024_1635_MOESM1_ESM.pdf]

nature microbiology

Supplementary information https://doi.org/10.1038/s41564-024-01635-8

Longitudinal multi-omics analysis of host microbiome architecture and immune responses during short-term spaceflight

In the format provided by the authors and unedited

Supplementary Figure 1: Top 10 bacterial genera identified by site by GTDB in metagenomic sequencing. A) Raw alignment data. B) Decontaminated reads.

Supplementary Figure 2: Top 10 bacterial genera identified by site by GTDB in metatranscriptomic sequencing. A) Raw alignment data. B) Decontaminated reads.

Supplementary Figure 3: Top 10 viral genera identified by site by GenBank alignment in metagenomic sequencing. A) Raw alignment data. B) Decontaminated reads.

Supplementary Figure 4: Top 10 viral genera identified by site by GenBank alignment in metatranscriptomic sequencing. A) Raw alignment data. B) Decontaminated reads.

Supplementary Figure 5: Top 10 genera identified by site by Kraken2 in metagenomic sequencing. A) Raw alignment data. B) Decontaminated reads.

Supplementary Figure 6: Top 10 genera identified by site by Kraken2 in metatranscriptomic sequencing. A) Raw alignment data. B) Decontaminated reads.

Supplementary Figure 7: Top 25 bacterial genera identified by site by GTDB in (A) metagenomic sequencing and (B) metatranscriptomic sequencing in the ground control and mid-flight capsule swabs.

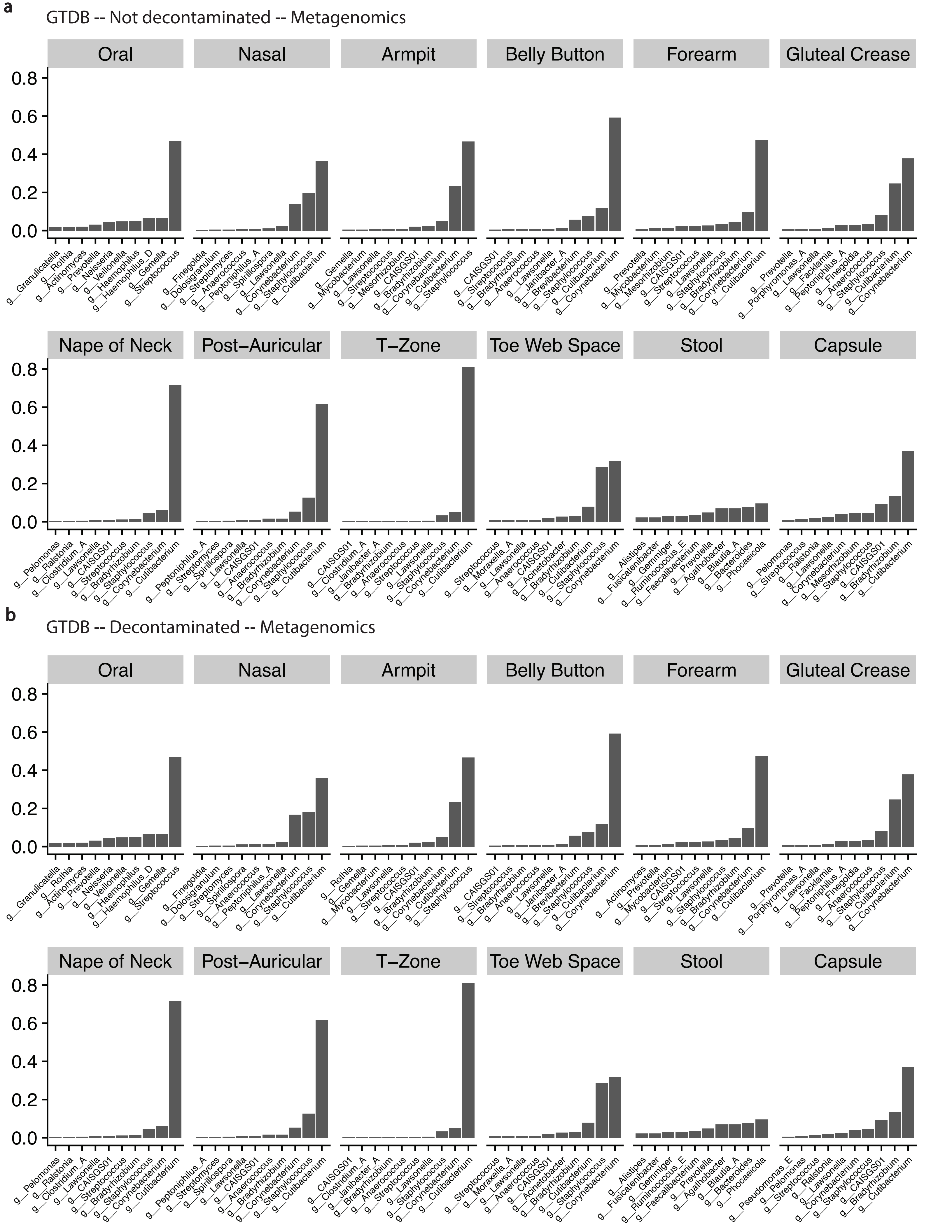
a
GTDB -- Not decontaminated -- Metagenomics
Oral
Nasal
Armpit
Belly Button
Forearm
Gluteal Crease
Nape of Neck
Post–Auricular
T–Zone
Toe Web Space
Stool
Capsule
b
GTDB -- Decontaminated -- Metagenomics
Oral
Nasal
Armpit
Belly Button
Forearm
Gluteal Crease
Nape of Neck
Post–Auricular
T–Zone
Toe Web Space
Stool
Capsule

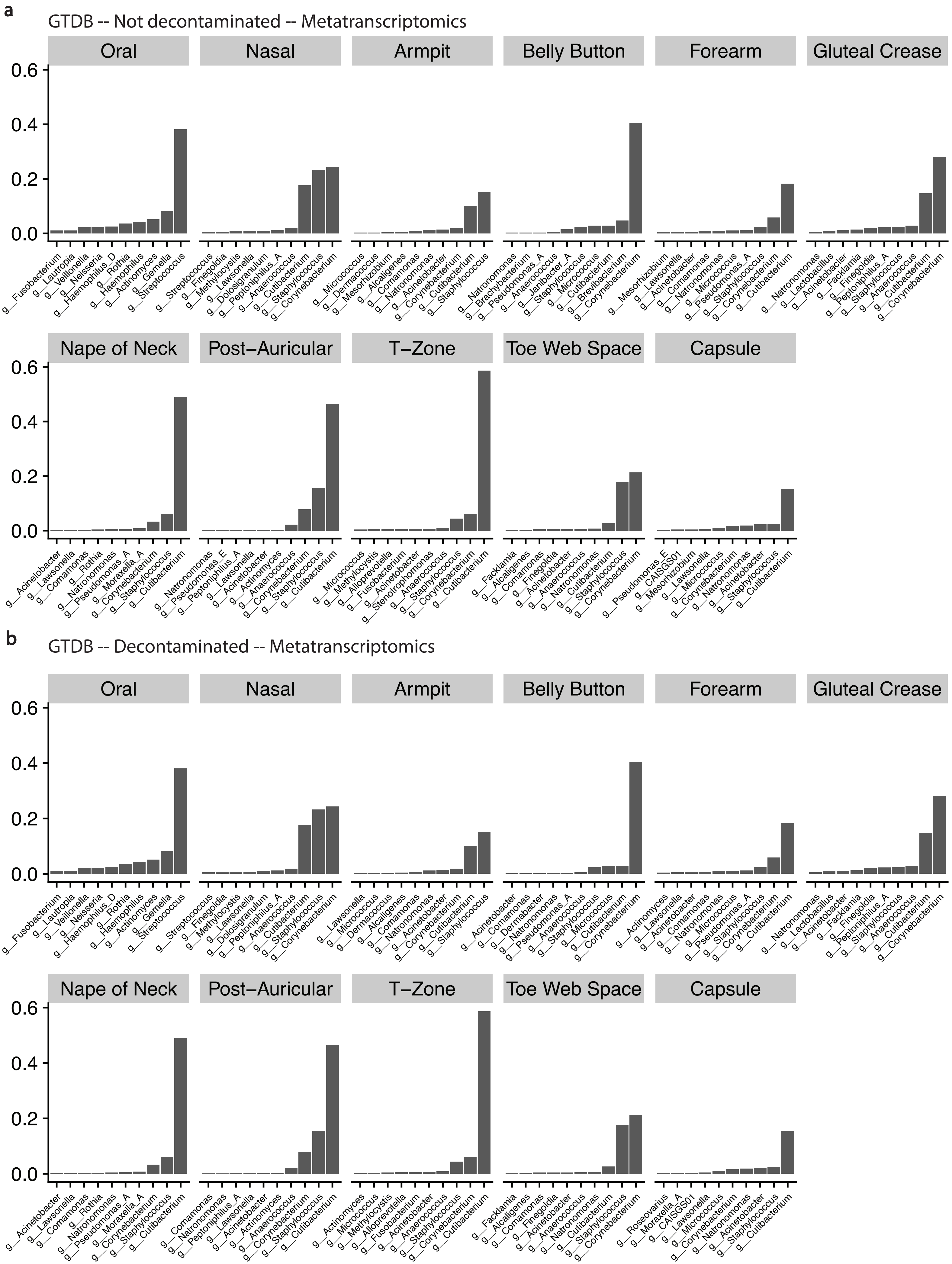
a
GTDB -- Not decontaminated -- Metatranscriptomics
Oral
Nasal
Armpit
Belly Button
Forearm
Gluteal Crease
Nape of Neck
Post−Auricular
T−Zone
Toe Web Space
Capsule
0.0
0.2
0.4
0.6
g__Fusobacterium g__Lautropia g__Veillonella g__Neisseria g__Haemophilus_D g__Rothia g__Haemophilus g__Actinomyces g__Gemella g__Streptococcus
g__Streptococcus g__Finegoldia g__Methylocystis g__Lawsonella g__Dolosigranulum g__Peptoniphilus_A g__Anaerococcus g__Cutibacterium g__Staphylococcus g__Corynebacterium
g__Micrococcus g__Dermacoccus g__Mesorhizobium g__Alcaligenes g__Comamonas g__Natronomonas g__Acinetobacter g__Corynebacterium g__Cutibacterium g__Staphylococcus
g__Natronomonas g__Brachybacterium g__Pseudomonas_A g__Anaerococcus g__Janibacter_A g__Staphylococcus g__Micrococcus g__Cutibacterium g__Brevibacterium g__Corynebacterium
g__Mesorhizobium g__Lawsonella g__Acinetobacter g__Comamonas g__Natronomonas g__Micrococcus g__Pseudomonas_A g__Staphylococcus g__Corynebacterium g__Cutibacterium
g__Natronomonas g__Lactobacillus g__Acinetobacter g__Facklamia g__Finegoldia g__Peptoniphilus_A g__Staphylococcus g__Anaerococcus g__Cutibacterium g__Corynebacterium
g__Acinetobacter g__Lawsonella g__Comamonas g__Rothia g__Natronomonas g__Pseudomonas_A g__Moraxella_A g__Corynebacterium g__Staphylococcus g__Cutibacterium
g__Natronomonas g__Pseudomonas_E g__Peptoniphilus_A g__Lawsonella g__Acinetobacter g__Actinomyces g__Anaerococcus g__Corynebacterium g__Staphylococcus g__Cutibacterium
g__Micrococcus g__Methylocystis g__Alloprevotella g__Fusobacterium g__Acinetobacter g__Stenotrophomonas g__Anaerococcus g__Staphylococcus g__Corynebacterium g__Cutibacterium
g__Facklamia g__Alcaligenes g__Comamonas g__Finegoldia g__Acinetobacter g__Anaerococcus g__Natronomonas g__Cutibacterium g__Staphylococcus g__Corynebacterium
g__Pseudomonas_E g__CAISGS01 g__Mesorhizobium g__Lawsonella g__Micrococcus g__Corynebacterium g__Natronomonas g__Acinetobacter g__Staphylococcus g__Cutibacterium
b
GTDB -- Decontaminated -- Metatranscriptomics
Oral
Nasal
Armpit
Belly Button
Forearm
Gluteal Crease
Nape of Neck
Post−Auricular
T−Zone
Toe Web Space
Capsule
0.0
0.2
0.4
0.6
g__Fusobacterium g__Lautropia g__Veillonella g__Neisseria g__Haemophilus_D g__Rothia g__Haemophilus g__Actinomyces g__Gemella g__Streptococcus
g__Streptococcus g__Finegoldia g__Methylocystis g__Lawsonella g__Dolosigranulum g__Peptoniphilus_A g__Anaerococcus g__Cutibacterium g__Staphylococcus g__Corynebacterium
g__Lawsonella g__Micrococcus g__Dermacoccus g__Alcaligenes g__Comamonas g__Natronomonas g__Acinetobacter g__Corynebacterium g__Cutibacterium g__Staphylococcus
g__Acinetobacter g__Comamonas g__Dermabacter g__Natronomonas g__Pseudomonas_A g__Anaerococcus g__Staphylococcus g__Micrococcus g__Cutibacterium g__Corynebacterium
g__Actinomyces g__Lawsonella g__Acinetobacter g__Comamonas g__Natronomonas g__Micrococcus g__Pseudomonas_A g__Staphylococcus g__Corynebacterium g__Cutibacterium
g__Natronomonas g__Lactobacillus g__Acinetobacter g__Facklamia g__Finegoldia g__Peptoniphilus_A g__Staphylococcus g__Anaerococcus g__Cutibacterium g__Corynebacterium
g__Acinetobacter g__Lawsonella g__Comamonas g__Rothia g__Natronomonas g__Pseudomonas_A g__Moraxella_A g__Corynebacterium g__Staphylococcus g__Cutibacterium
g__Comamonas g__Natronomonas g__Peptoniphilus_A g__Lawsonella g__Acinetobacter g__Actinomyces g__Anaerococcus g__Corynebacterium g__Staphylococcus g__Cutibacterium
g__Actinomyces g__Micrococcus g__Methylocystis g__Alloprevotella g__Fusobacterium g__Acinetobacter g__Anaerococcus g__Staphylococcus g__Corynebacterium g__Cutibacterium
g__Facklamia g__Alcaligenes g__Comamonas g__Finegoldia g__Acinetobacter g__Anaerococcus g__Natronomonas g__Cutibacterium g__Staphylococcus g__Corynebacterium
g__Roseovarius g__Moraxella_A g__CAISGS01 g__Lawsonella g__Micrococcus g__Corynebacterium g__Natronomonas g__Acinetobacter g__Staphylococcus g__Cutibacterium

a GenBank Viral-- Not decontaminated -- Metagenomics

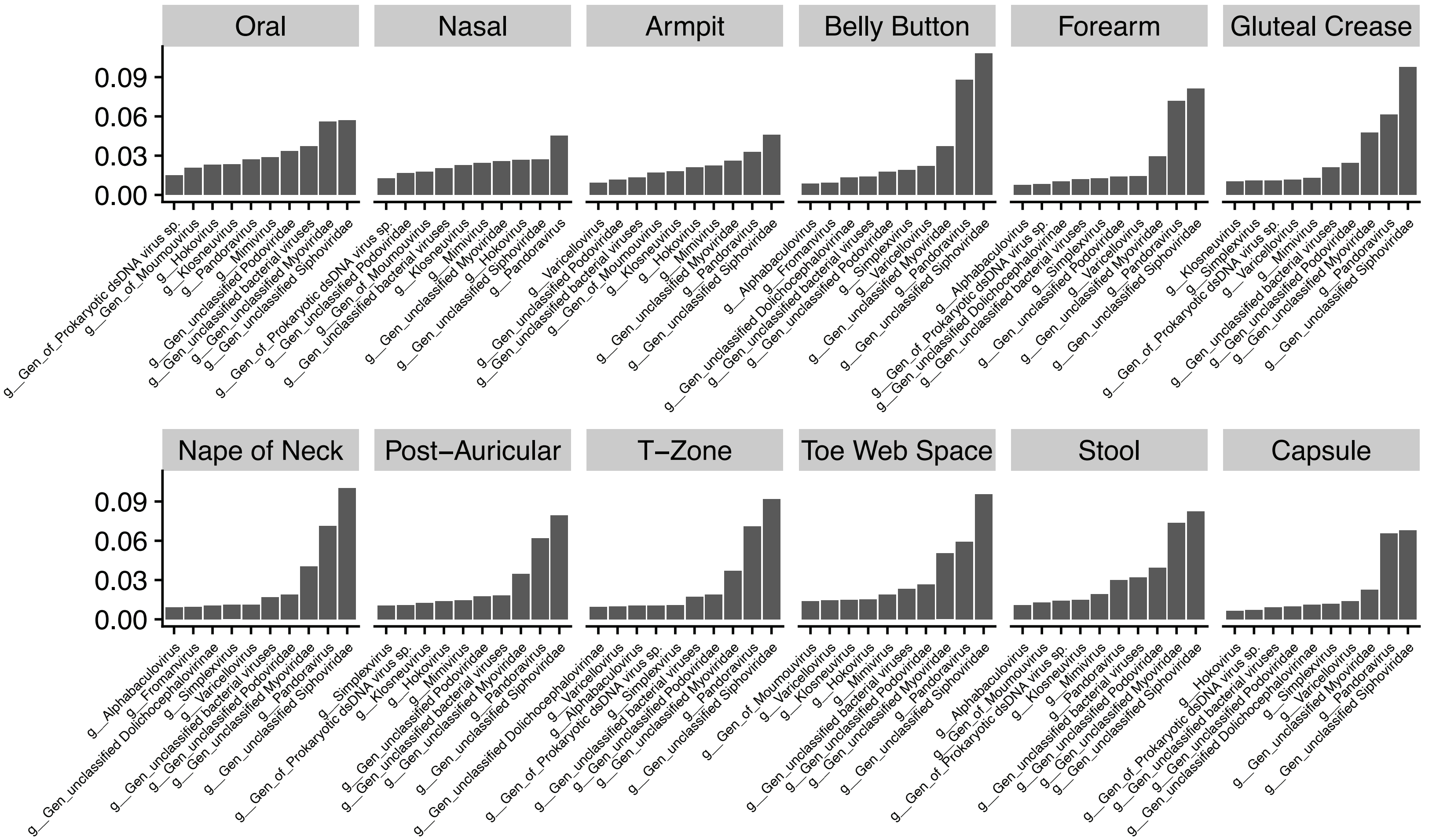

b GenBank Viral -- Decontaminated -- Metagenomics

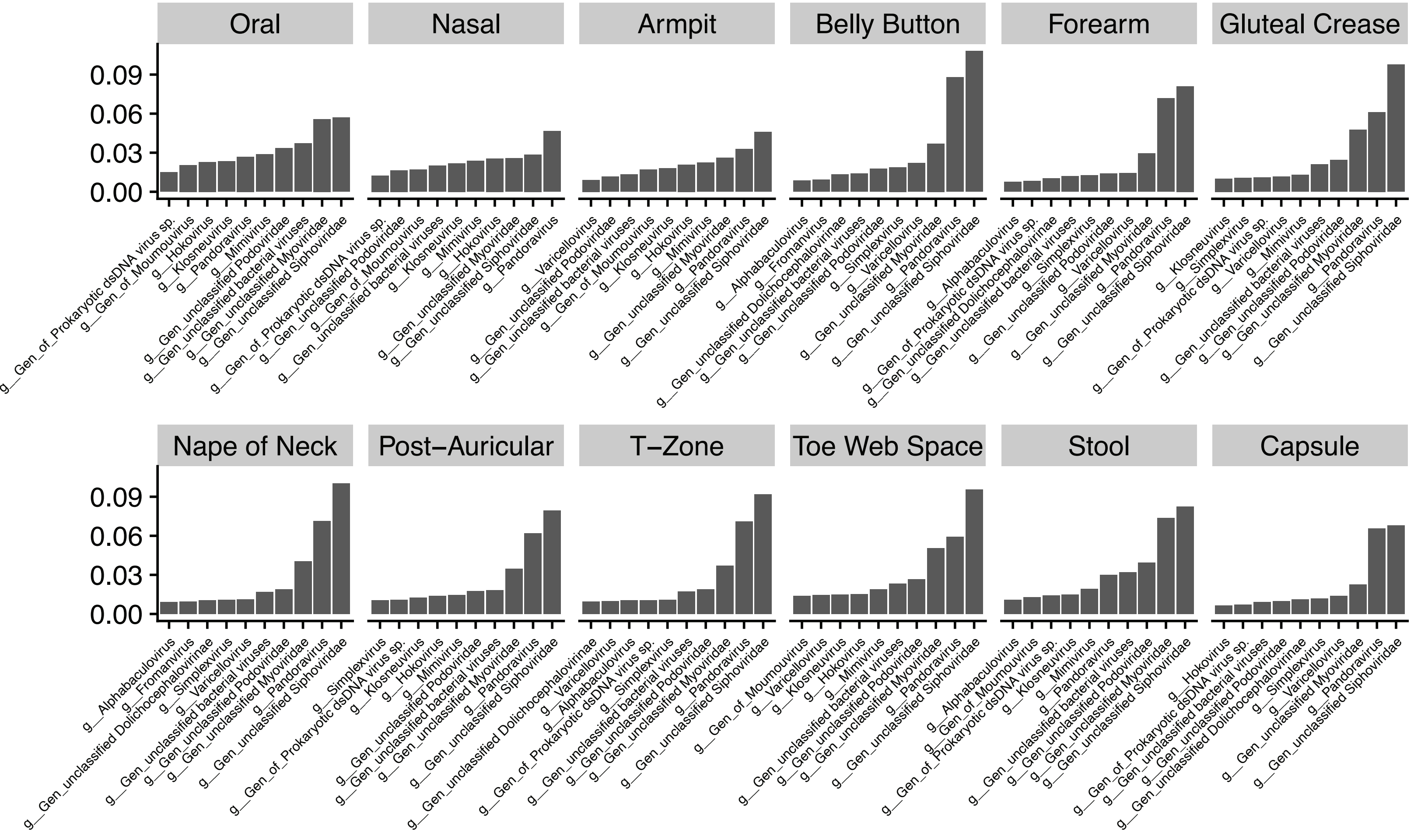

a

GenBank Viral-- Not decontaminated -- Metatranscriptomics

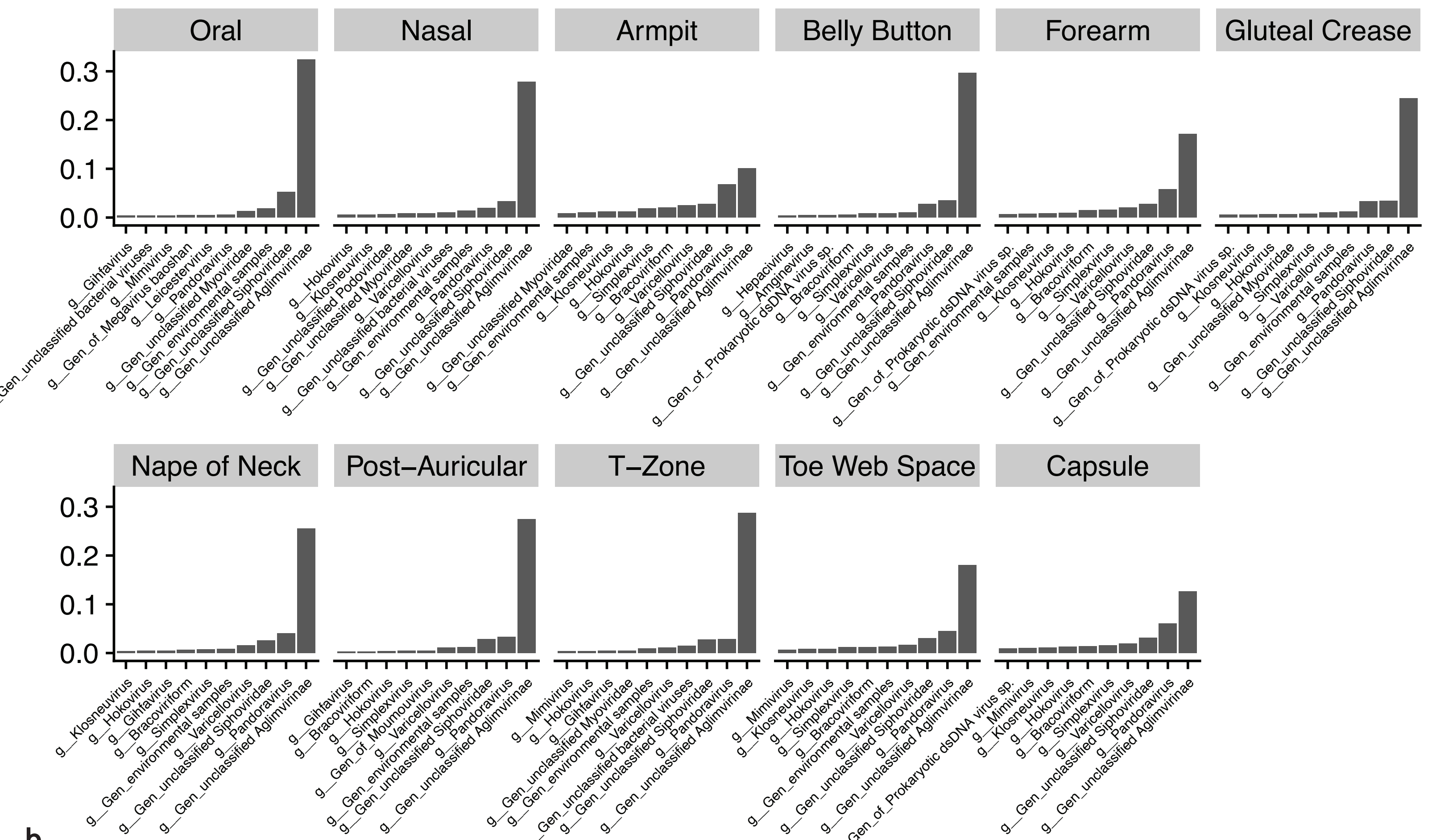

b

GenBank Viral -- Decontaminated -- Metatranscriptomics

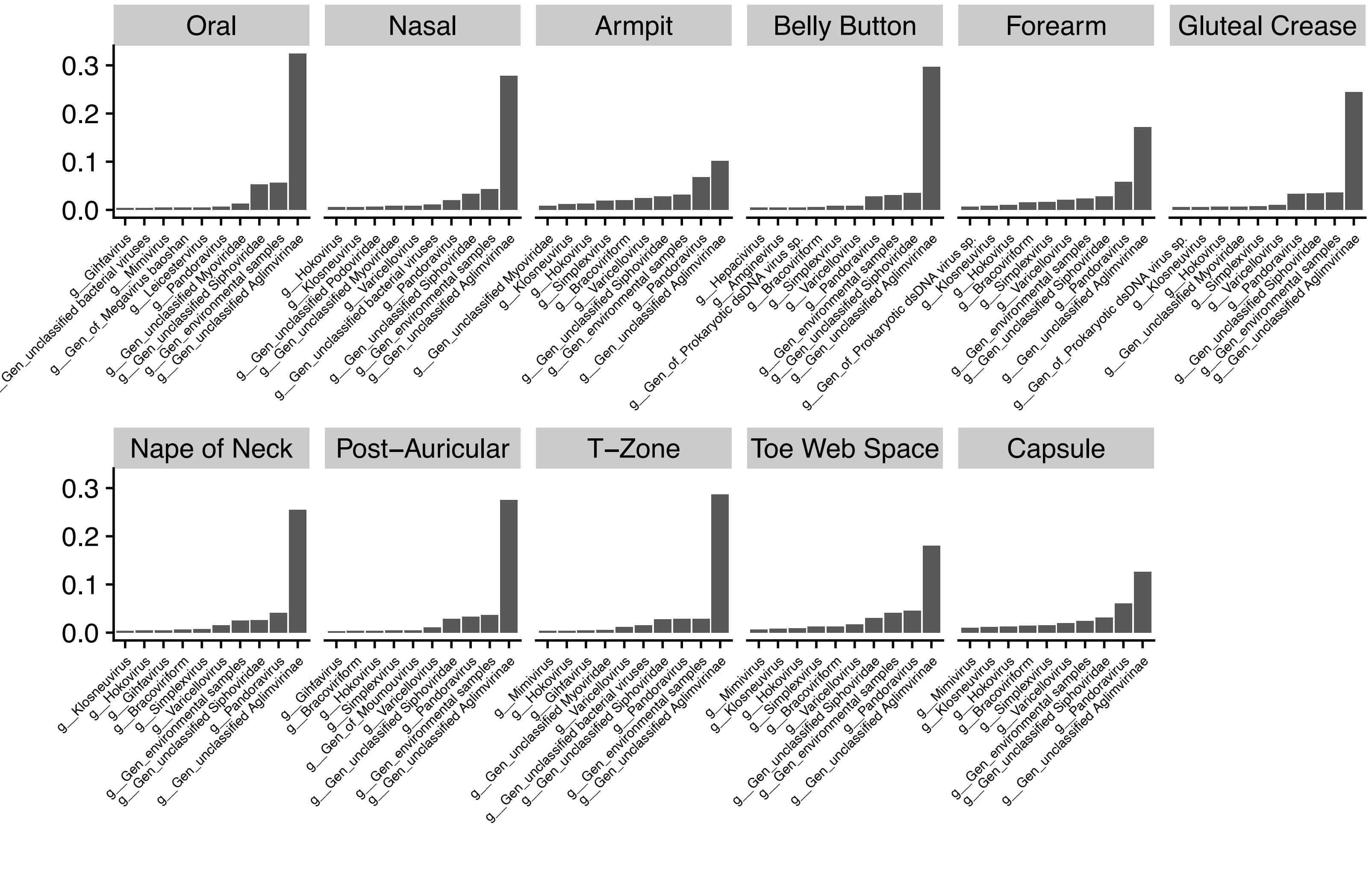

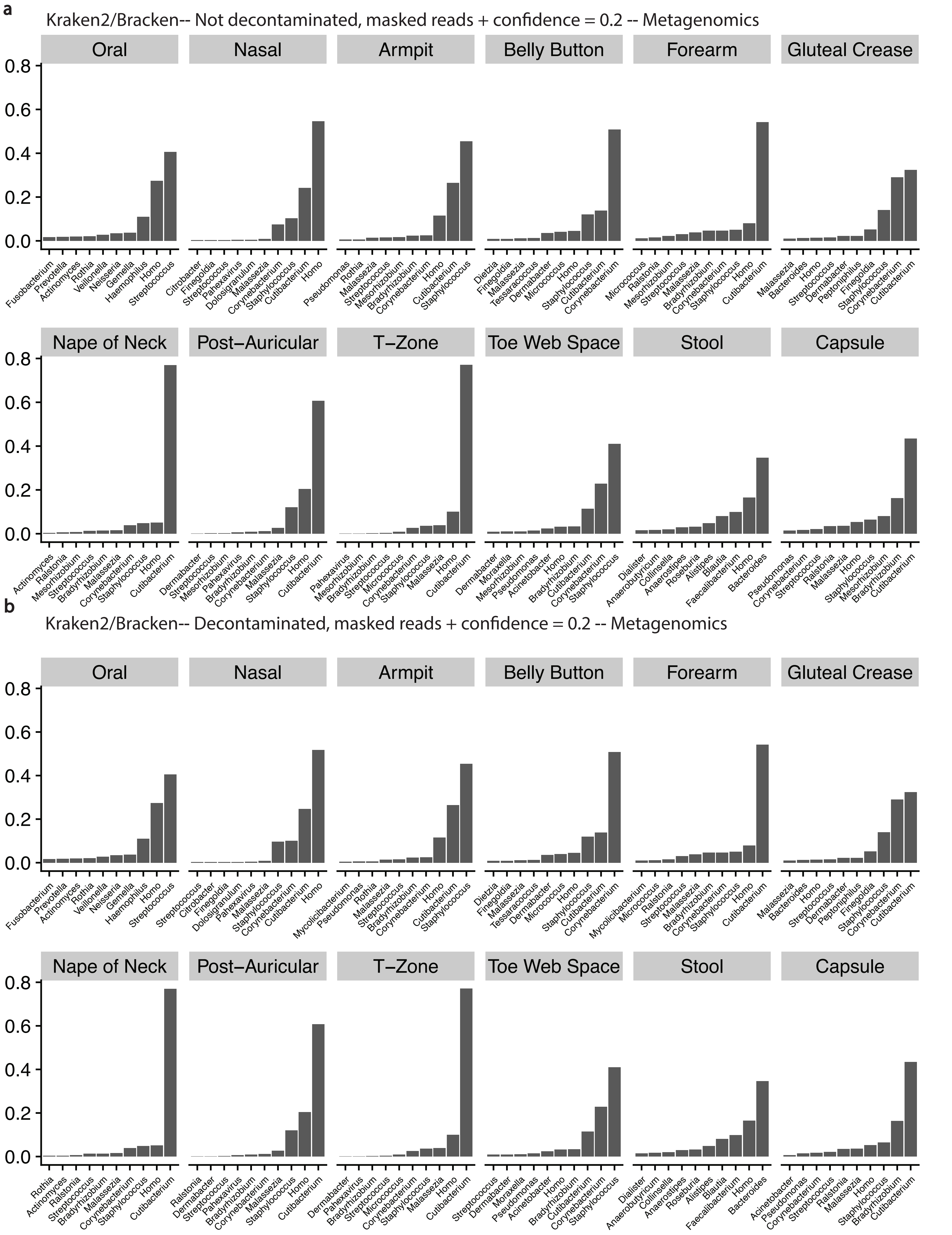
a
Kraken2/Bracken-- Not decontaminated, masked reads + confidence = 0.2 -- Metagenomics
Oral
Nasal
Armpit
Belly Button
Forearm
Gluteal Crease
0.8
0.6
0.4
0.2
0.0
Fusobacterium Prevotella Actinomyces Rothia Veillonella Neisseria Gemella Haemophilus Homo Streptococcus
Citrobacter Finegoldia Streptococcus Pahexavirus Dolosigranulum Malassezia Corynebacterium Staphylococcus Cutibacterium Homo
Pseudomonas Rothia Malassezia Streptococcus Mesorhizobium Bradyrhizobium Corynebacterium Homo Cutibacterium Staphylococcus
Dietzia Finegoldia Malassezia Tessaracoccus Dermabacter Micrococcus Homo Staphylococcus Cutibacterium Corynebacterium
Micrococcus Ralstonia Mesorhizobium Streptococcus Malassezia Bradyrhizobium Corynebacterium Staphylococcus Homo Cutibacterium
Malassezia Bacteroides Homo Streptococcus Dermabacter Peptoniphilus Finegoldia Staphylococcus Corynebacterium Cutibacterium
Nape of Neck
Post−Auricular
T−Zone
Toe Web Space
Stool
Capsule
Actinomyces Ralstonia Mesorhizobium Streptococcus Bradyrhizobium Malassezia Corynebacterium Staphylococcus Homo Cutibacterium
Dermabacter Streptococcus Mesorhizobium Pahexavirus Bradyrhizobium Corynebacterium Malassezia Staphylococcus Homo Cutibacterium
Pahexavirus Mesorhizobium Bradyrhizobium Streptococcus Micrococcus Corynebacterium Staphylococcus Malassezia Homo Cutibacterium
Dermabacter Moraxella Mesorhizobium Pseudomonas Acinetobacter Homo Bradyrhizobium Cutibacterium Corynebacterium Staphylococcus
Dialister Anaerobutyricum Collinsella Anaerostipes Roseburia Alistipes Blautia Faecalibacterium Homo Bacteroides
Pseudomonas Corynebacterium Streptococcus Ralstonia Malassezia Homo Staphylococcus Mesorhizobium Bradyrhizobium Cutibacterium
b
Kraken2/Bracken-- Decontaminated, masked reads + confidence = 0.2 -- Metagenomics
Oral
Nasal
Armpit
Belly Button
Forearm
Gluteal Crease
Fusobacterium Prevotella Actinomyces Rothia Veillonella Neisseria Gemella Haemophilus Homo Streptococcus
Streptococcus Citrobacter Finegoldia Dolosigranulum Pahexavirus Malassezia Staphylococcus Corynebacterium Cutibacterium Homo
Mycolicibacterium Pseudomonas Rothia Malassezia Streptococcus Bradyrhizobium Corynebacterium Homo Cutibacterium Staphylococcus
Dietzia Finegoldia Malassezia Tessaracoccus Dermabacter Micrococcus Homo Staphylococcus Cutibacterium Corynebacterium
Mycolicibacterium Micrococcus Ralstonia Streptococcus Malassezia Bradyrhizobium Corynebacterium Staphylococcus Homo Cutibacterium
Malassezia Bacteroides Homo Streptococcus Dermabacter Peptoniphilus Finegoldia Staphylococcus Corynebacterium Cutibacterium
Nape of Neck
Post−Auricular
T−Zone
Toe Web Space
Stool
Capsule
Rothia Actinomyces Ralstonia Streptococcus Bradyrhizobium Malassezia Corynebacterium Staphylococcus Homo Cutibacterium
Ralstonia Dermabacter Streptococcus Pahexavirus Bradyrhizobium Corynebacterium Malassezia Staphylococcus Homo Cutibacterium
Dermabacter Pahexavirus Bradyrhizobium Streptococcus Micrococcus Corynebacterium Staphylococcus Malassezia Homo Cutibacterium
Streptococcus Dermabacter Moraxella Pseudomonas Acinetobacter Homo Bradyrhizobium Cutibacterium Corynebacterium Staphylococcus
Dialister Anaerobutyricum Collinsella Anaerostipes Roseburia Alistipes Blautia Faecalibacterium Homo Bacteroides
Acinetobacter Pseudomonas Corynebacterium Streptococcus Ralstonia Malassezia Homo Staphylococcus Bradyrhizobium Cutibacterium

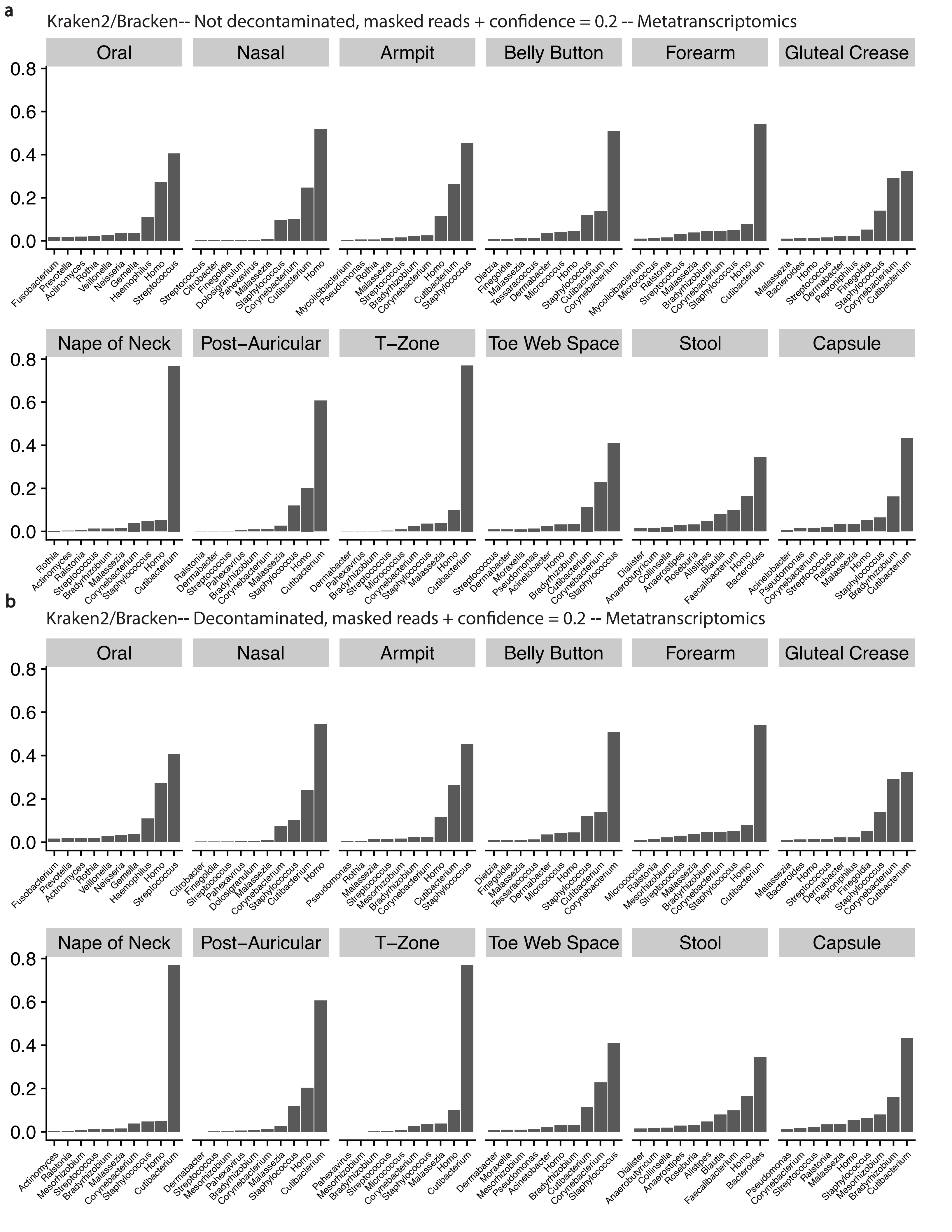
a
Kraken2/Bracken-- Not decontaminated, masked reads + confidence = 0.2 -- Metatranscriptomics
Oral
Nasal
Armpit
Belly Button
Forearm
Gluteal Crease
Nape of Neck
Post−Auricular
T−Zone
Toe Web Space
Stool
Capsule
0.0
0.2
0.4
0.6
0.8
Fusobacterium Prevotella Actinomyces Rothia Veillonella Neisseria Gemella Haemophilus Homo Streptococcus
Streptococcus Citrobacter Finegoldia Dolosigranulum Pahexavirus Malassezia Staphylococcus Corynebacterium Cutibacterium Homo
Mycolicibacterium Pseudomonas Rothia Malassezia Streptococcus Bradyrhizobium Corynebacterium Homo Cutibacterium Staphylococcus
Dietzia Finegoldia Malassezia Tessaracoccus Dermabacter Micrococcus Homo Staphylococcus Cutibacterium Corynebacterium
Mycolicibacterium Micrococcus Ralstonia Streptococcus Malassezia Bradyrhizobium Corynebacterium Staphylococcus Homo Cutibacterium
Malassezia Bacteroides Homo Streptococcus Dermabacter Peptoniphilus Finegoldia Staphylococcus Corynebacterium Cutibacterium
Rothia Actinomyces Ralstonia Streptococcus Bradyrhizobium Malassezia Corynebacterium Staphylococcus Homo Cutibacterium
Ralstonia Dermabacter Streptococcus Pahexavirus Bradyrhizobium Corynebacterium Malassezia Staphylococcus Homo Cutibacterium
Dermabacter Pahexavirus Bradyrhizobium Streptococcus Micrococcus Corynebacterium Staphylococcus Malassezia Homo Cutibacterium
Streptococcus Dermabacter Moraxella Pseudomonas Acinetobacter Homo Bradyrhizobium Cutibacterium Corynebacterium Staphylococcus
Dialister Anaerobutyricum Collinsella Anaerostipes Roseburia Alistipes Blautia Faecalibacterium Homo Bacteroides
Acinetobacter Pseudomonas Corynebacterium Streptococcus Ralstonia Malassezia Homo Staphylococcus Bradyrhizobium Cutibacterium
b
Kraken2/Bracken-- Decontaminated, masked reads + confidence = 0.2 -- Metatranscriptomics
Oral
Nasal
Armpit
Belly Button
Forearm
Gluteal Crease
Nape of Neck
Post−Auricular
T−Zone
Toe Web Space
Stool
Capsule
0.0
0.2
0.4
0.6
0.8
Fusobacterium Prevotella Actinomyces Rothia Veillonella Neisseria Gemella Haemophilus Homo Streptococcus
Citrobacter Finegoldia Streptococcus Pahexavirus Dolosigranulum Malassezia Corynebacterium Staphylococcus Cutibacterium Homo
Pseudomonas Rothia Malassezia Streptococcus Mesorhizobium Bradyrhizobium Corynebacterium Homo Cutibacterium Staphylococcus
Dietzia Finegoldia Malassezia Tessaracoccus Dermabacter Micrococcus Homo Staphylococcus Cutibacterium Corynebacterium
Micrococcus Ralstonia Mesorhizobium Streptococcus Malassezia Bradyrhizobium Corynebacterium Staphylococcus Homo Cutibacterium
Malassezia Bacteroides Homo Streptococcus Dermabacter Peptoniphilus Finegoldia Staphylococcus Corynebacterium Cutibacterium
Actinomyces Ralstonia Mesorhizobium Streptococcus Bradyrhizobium Malassezia Corynebacterium Staphylococcus Homo Cutibacterium
Dermabacter Streptococcus Mesorhizobium Pahexavirus Bradyrhizobium Corynebacterium Malassezia Staphylococcus Homo Cutibacterium
Pahexavirus Mesorhizobium Bradyrhizobium Streptococcus Micrococcus Corynebacterium Staphylococcus Malassezia Homo Cutibacterium
Dermabacter Moraxella Mesorhizobium Pseudomonas Acinetobacter Homo Bradyrhizobium Cutibacterium Corynebacterium Staphylococcus
Dialister Anaerobutyricum Collinsella Anaerostipes Roseburia Alistipes Blautia Faecalibacterium Homo Bacteroides
Pseudomonas Corynebacterium Streptococcus Ralstonia Malassezia Homo Staphylococcus Mesorhizobium Bradyrhizobium Cutibacterium
