# Supplementary material for: Longitudinal multi-omics analysis of host microbiome architecture and immune responses during short-term spaceflight
Source: Nat Microbiol. 2024 Jun 11;9(7):1661–75. doi: 10.1038/s41564-024-01635-8 (PMC11222149; doi:10.1038/s41564-024-01635-8)
Supplement: Supplementary file 2 — Reporting Summary [file 41564_2024_1635_MOESM2_ESM.pdf]

nature portfolio

Corresponding author(s): Christopher E. Mason

Last updated by author(s): January 10th, 2024

Reporting Summary

Nature Portfolio wishes to improve the reproducibility of the work that we publish. This form provides structure for consistency and transparency in reporting. For further information on Nature Portfolio policies, see our Editorial Policies and the Editorial Policy Checklist.

Statistics

For all statistical analyses, confirm that the following items are present in the figure legend, table legend, main text, or Methods section.

n/a	Confirmed	
☐	☒	The exact sample size (*n*) for each experimental group/condition, given as a discrete number and unit of measurement
☐	☒	A statement on whether measurements were taken from distinct samples or whether the same sample was measured repeatedly
☐	☒	The statistical test(s) used AND whether they are one- or two-sided<br>*Only common tests should be described solely by name; describe more complex techniques in the Methods section.*
☐	☒	A description of all covariates tested
☐	☒	A description of any assumptions or corrections, such as tests of normality and adjustment for multiple comparisons
☐	☒	A full description of the statistical parameters including central tendency (e.g. means) or other basic estimates (e.g. regression coefficient) AND variation (e.g. standard deviation) or associated estimates of uncertainty (e.g. confidence intervals)
☐	☒	For null hypothesis testing, the test statistic (e.g. *F*, *t*, *r*) with confidence intervals, effect sizes, degrees of freedom and *P* value noted<br>*Give P values as exact values whenever suitable.*
☒	☐	For Bayesian analysis, information on the choice of priors and Markov chain Monte Carlo settings
☒	☐	For hierarchical and complex designs, identification of the appropriate level for tests and full reporting of outcomes
☒	☐	Estimates of effect sizes (e.g. Cohen's *d*, Pearson's *r*), indicating how they were calculated

Our web collection on statistics for biologists contains articles on many of the points above.

Software and code

Policy information about availability of computer code

Data collection	No software was used for data collection.
Data analysis	Code availability:<br>Code used to generate Figures and analyses from this project is available at https://github.com/eliah-o/inspiration4-omics.<br><br>Software:<br>bbtools (v38.92)XTree (v0.92i) Kraken2 (v2.l.2) bracken (v2.6.2) vegan (v2.6.2) art_illumina (v2.3.7) MetaSPAdes (v3.14.3) MetaQUAST (vS.0.2) Bowtie2 (v2.2.3) samtools (vl.0) CheckV (v0.8.1) Bakta (vl.5.1) MMseqs2 (vl3.4511) Diamond (v2.0.14) trimmomatic (v0.39) phanta (vl.1.0) metabat2 (V2.12.1) CheckM (V1.2) deRep (V3.2.2) MetaPhlan4 (v4.0.4) GTDB-tK (V2.1.1)

For manuscripts utilizing custom algorithms or software that are central to the research but not yet described in published literature, software must be made available to editors and reviewers. We strongly encourage code deposition in a community repository (e.g. GitHub). See the Nature Portfolio guidelines for submitting code & software for further information.

Data

Policy information about availability of data

All manuscripts must include a data availability statement. This statement should provide the following information, where applicable:

- Accession codes, unique identifiers, or web links for publicly available datasets
- A description of any restrictions on data availability
- For clinical datasets or third party data, please ensure that the statement adheres to our policy

The data that support this study is available at the NASA GeneLab/NASA Open Science Data Repository with the identifiers OSD-630 (https://doi.org/10.26030/cyfk-5f38), OSD-570 (https://doi.org/10.26030/41s1-j243), OSD-572 (https://doi.org/10.26030/x57b-4722) and OSD-573 and (https://doi.org/10.26030/x57b-4722). Additional processed datasets (gene catalogs, taxonomic and gene abundances) are available at https://figshare.com/ projects/Longitudinal_multi-omics_analysis_of_host_microbiome_architecture_and_immune_responses_during_short-term_spaceflight/176043. This Figshare repository additionally contains figures detailing the top most abundant taxa for each alignment algorithm before and after decontamination. Select data can be visualized online through the SOMA Data Explorer: https://soma.weill.cornell.edu. The GenBank viral database used was the most recent as of 2022-07-26. The GTDB database used was the 202 release. The MetaPhlan4 database was mpa_vJan21_CHOCOPhlAnSGB_202103. The Kraken2 database contained all NCBI listed taxa (bacteria, fungal, and viral genomes) in RefSeq, as of 2022-09-01. The Phanta database was the most recent as of 2022-08-01. The Bakta databases were the most recent as of 2022-08-18.

Human research participants

Policy information about studies involving human research participants and Sex and Gender in Research.

Reporting on sex and gender	Sex is reported in the manuscript.
Population characteristics	The crew member composition was of two races and ages ranged from 29-51.
Recruitment	Participants were recruited by SpaceX and mission commander Jared Isaacman; the authors did not have say in recruitment, as it was done prior to study onset. SpaceX and Mr. Isaacment recruited individuals with multiple demographic backgrounds fit for spaceflight. One won a contest, one was nominated by a US hospital, one won a raffle. Given that all individuals opted to take part in spaceflight, there be may associated self-selection biases.
Ethics oversight	This study was complete in accordance with appropriate ethical guidelines. All subjects were consented at an informed consent briefing (ICB) at SpaceX (Hawthorne, CA), and samples were collected and processed under the approval of the Institutional Review Board (IRB) at Weill Cornell Medicine, under Protocol 21-05023569. All crew members provided written informed consent for data and sample sharing.

Note that full information on the approval of the study protocol must also be provided in the manuscript.

Field-specific reporting

Please select the one below that is the best fit for your research. If you are not sure, read the appropriate sections before making your selection.

☒ Life sciences ☐ Behavioural & social sciences ☐ Ecological, evolutionary & environmental sciences

For a reference copy of the document with all sections, see nature.com/documents/nr-reporting-summary-flat.pdf

Life sciences study design

All studies must disclose on these points even when the disclosure is negative.

Sample size	The entire Inspiration4 crew was profiled, which was limited by the size of the Dragon capsule (n=4).
Data exclusions	No data has been excluded.
Replication	Replication tests were not performed; they are difficult as mission parameters could not be repeated.
Randomization	This is not relevant to the study as we were profiling the entire crew longitudinally (pre-flight, in-flight, and post-flight).
Blinding	Blinding was not possible because all subjects were astronauts in the same crew.

Reporting for specific materials, systems and methods

We require information from authors about some types of materials, experimental systems and methods used in many studies. Here, indicate whether each material, system or method listed is relevant to your study. If you are not sure if a list item applies to your research, read the appropriate section before selecting a response.

Materials & experimental systems

n/a	Involved in the study
☒	☐ Antibodies
☒	☐ Eukaryotic cell lines
☒	☐ Palaeontology and archaeology
☒	☐ Animals and other organisms
☒	☐ Clinical data
☒	☐ Dual use research of concern

Methods

n/a	Involved in the study
☒	☐ ChIP-seq
☒	☐ Flow cytometry
☒	☐ MRI-based neuroimaging
